# The New Coronavirus (SARS-CoV-2): A Comprehensive Review on Immunity and the Application of Bioinformatics and Molecular Modeling to the Discovery of Potential Anti-SARS-CoV-2 Agents

**DOI:** 10.3390/molecules25184086

**Published:** 2020-09-07

**Authors:** Gustavo R. Villas-Boas, Vanessa C. Rescia, Marina M. Paes, Stefânia N. Lavorato, Manoel F. de Magalhães-Filho, Mila S. Cunha, Rafael da C. Simões, Roseli B. de Lacerda, Renilson S. de Freitas-Júnior, Bruno H. da S. Ramos, Ana M. Mapeli, Matheus da S. T. Henriques, William R. de Freitas, Luiz A. F. Lopes, Luiz G. R. Oliveira, Jonatas G. da Silva, Saulo E. Silva-Filho, Ana P. S. da Silveira, Katyuscya V. Leão, Maria M. de S. Matos, Jamille S. Fernandes, Roberto K. N. Cuman, Francielli M. de S. Silva-Comar, Jurandir F. Comar, Luana do A. Brasileiro, Jussileide N. dos Santos, Silvia A. Oesterreich

**Affiliations:** 1 Research Group on Development of Pharmaceutical Products (P&DProFar), Center for Biological and Health Sciences, Federal University of Western Bahia, Rua Bertioga, 892, Morada Nobre II, Barreiras CEP 47810-059, BA, Brazil; vanessa.rescia@ufob.edu.br (V.C.R.); marinameirelles@ymail.com (M.M.P.); stefania.lavorato@ufob.edu.br (S.N.L.); manoel.magalhaes17@gmail.com (M.F.d.M.-F.); milacunha035@gmail.com (M.S.C.); rafael.simoes@ufob.edu.br (R.d.C.S.); 2 Department of Pharmacology of the Biological Sciences Center, Federal University of Paraná, Jardim das Américas, Caixa. postal 19031, Curitiba CEP 81531-990, PR, Brazil; boerngen@hotmail.com; 3 Clinical Health is Life-Integrated Health Center, Rua dos Andrades, 99, Barreirinhas, Barreiras CEP 47810-689, BA, Brazil; renilsonfreitas@gmail.com; 4 Institute of the Spine and Pain Clinic, Rua Dr. Renato Gonçalves, 108, Renato Gonçalves, Barreiras CEP 47806-021, BA, Brazil; Drbrunoramos@hotmail.com; 5 Research Group on Biomolecules and Catalyze, Center for Biological and Health Sciences, Federal University of Western Bahia, Rua Bertioga, 892, Morada Nobre II, Barreiras CEP 47810-059, BA, Brazil; mmapeli@ufob.edu.br; 6 Laboratory of Pharmacology of Toxins (LabTox), Graduate Program in Pharmacology and Medicinal Chemistry (PPGFQM), Institute of Biomedical Sciences (ICB) Federal University of Rio de Janeiro (UFRJ), Avenida Carlos Chagas Filho, 373, Cidade Universitária, Rio de Janeiro CEP 21941-590, RJ, Brazil; matheussth@hotmail.com; 7 Research Group on Biodiversity and Health (BIOSA), Center for Training in Health Sciences, Federal University of Southern Bahia, Praça Joana Angélica, 58, São José, Teixeira de Freitas, Teixeira de Freitas CEP 45988-058, Brazil; william.freitas@ufsb.edu.br; 8 University Hospital of the Federal University of Grande Dourados (HU-UFGD), Federal University of Grande Dourados, Rua Ivo Alves da Rocha, 558, Altos do Indaiá, Dourados CEP 79823-501, MS, Brazil; luiz.lopes@ebserh.gov.br; 9 Nucleus of Studies on Infectious Agents and Vectors (Naive), Federal University of Western Bahia, Rua Bertioga, 892, Morada Nobre II, Barreiras CEP 47810-059, BA, Brazil; luiz.oliveira@ufob.edu.br; 10 Federal University of Western Bahia, Rua Bertioga, 892, Morada Nobre II, Barreiras CEP 47810-059, BA, Brazil; gomes.jonatas@gmail.com (J.G.d.S.); kleao@ufob.edu.br (K.V.L.); sf.jamille@gmail.com (J.S.F.); 11 Pharmaceutical Sciences, Food and Nutrition College, Federal University of Mato Grosso do Sul, Avenida Costa e Silva, s/nº, Bairro Universitário, Campo Grande CEP 79070-900, MS, Brazil; saulo.e@ufms.br; 12 Faculty of Biological and Health Sciences, University Center Unigran Capital, Rua Balbina de Matos, 2121, Jd. University, Dourados CEP 79.824-900, MS, Brazil; anapaulastefanello@hotmail.com; 13 Health Sciences at ABC Health University Center, Avenida Príncipe de Gales, 667, Bairro Princípe de Gales, Santo André CEP 09060-870, SP, Brazil; madatos2906@gmail.com; 14 Department of Pharmacology and Therapeutics, State University of Maringá, Avenida Colombo, nº 5790, Jardim Universitário, Maringá CEP 87020-900, PR, Brazil; rkncuman@uem.br (R.K.N.C.); franciellimss@gmail.com (F.M.d.S.S.-C.); 15 Department of Biochemistry, State University of Maringá, Avenida Colombo, nº 5790, Jardim Universitário, Maringá CEP 87020-900, PR, Brazil; jfcomar@uem.br; 16 Nacional Cancer Institute (INCA), Rua Visconde de Santa Isabel, 274, Rio de Janeiro CEP 20560-121, RJ, Brazil; luana.brasileiro@inca.gov.br; 17 Natu Flora, Rua José Rocha, n° 239, Barreiras CEP 47.800-184, BA, Brazil; leide-neves@hotmail.com; 18 Faculty of Health Sciences, Federal University of Grande Dourados, Dourados Rodovia Dourados, Itahum Km 12, Cidade Universitaria, Caixa postal 364, Dourados CEP 79804-970, Mato Grosso do Sul, Brazil; silviaoesterreich@gmail.com

**Keywords:** SARS-CoV-2, drug discovery, bioinformatics, molecular modeling, innate immunity, treatment, infectious disease

## Abstract

On March 11, 2020, the World Health Organization (WHO) officially declared the outbreak caused by the new coronavirus (SARS-CoV-2) a pandemic. The rapid spread of the disease surprised the scientific and medical community. Based on the latest reports, news, and scientific articles published, there is no doubt that the coronavirus has overloaded health systems globally. Practical actions against the recent emergence and rapid expansion of the SARS-CoV-2 require the development and use of tools for discovering new molecular anti-SARS-CoV-2 targets. Thus, this review presents bioinformatics and molecular modeling strategies that aim to assist in the discovery of potential anti-SARS-CoV-2 agents. Besides, we reviewed the relationship between SARS-CoV-2 and innate immunity, since understanding the structures involved in this infection can contribute to the development of new therapeutic targets. Bioinformatics is a technology that assists researchers in coping with diseases by investigating genetic sequencing and seeking structural models of potential molecular targets present in SARS-CoV2. The details provided in this review provide future points of consideration in the field of virology and medical sciences that will contribute to clarifying potential therapeutic targets for anti-SARS-CoV-2 and for understanding the molecular mechanisms responsible for the pathogenesis and virulence of SARS-CoV-2.

## 1. Introduction

An unprecedented pneumonia outbreak of unknown etiology emerged in December 2019 in the city of Wuhan (Hubei Province, China) [1]. Affected patients were epidemiologically linked to the Huanan Seafood Wholesale Market and were identified using a surveillance mechanism for “pneumonia of unknown etiology” by local hospitals [2]. One month later, scientists isolated a novel coronavirus (SARS-CoV-2), which was reported to be a member of the β subfamily of coronaviruses [3,4,5]. The World Health Organization (WHO) introduced the name COVID-19 for the disease caused by this etiological agent [6]. Previous studies have shown that a large number of severe acute respiratory syndrome related coronaviruses (SARSr-CoVs) exist in bats [7,8,9], and there is serological evidence, that some bat SARSr-CoVs have the potential to infect humans [7,10]. A recent genetic analysis of full-length genome sequences obtained from some patients at the early stage of the outbreak revealed that the sequences are almost identical, sharing a 79.6% sequence identity to SARS-CoV and SARS-CoV-2 is 96% identical at a whole-genome level to a bat coronavirus [7,11]. This indicates bats as the likely origin and suggested the high possibility of animal-to-human transmission [11]. Subsequently, human-to-human transmission was confirmed.

**Figure 1:**
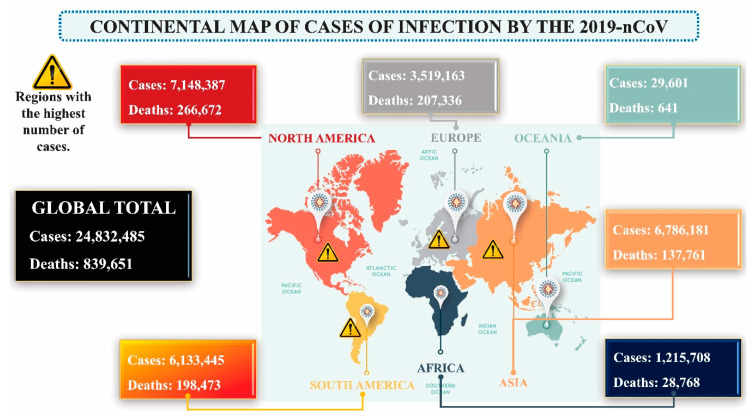
Continental map of SARS-CoV-2 infection cases. Data were collected on 28.08.2020 [15]. Designed by Freepik.

Thus, the number of COVID-19 cases in Wuhan, the disease epicenter, quickly climbed, and besides China, there are now more than 200 countries with confirmed COVID-19 cases. [12]. Figure 1 shows the number of infected patients and deaths caused by COVID-19 until the end of August 2020. Regarding the SARS-CoV-2 outbreak on 30 January 2020, WHO declared the disease to be a Public Health Emergency of International Concern (PHEIC) [4,13]. To date, the typical clinical symptoms of these patients are fever, dry cough, sore throat, breathing difficulties (dyspnoea), headache and pneumonia [7,14]. Given the exponential growth of SARS-CoV-2 infection cases, the development of tools that contribute to discovering of new therapeutic targets against the virus is crucial to prevent more SARS-CoV-2 related deaths.

Currently, in the absence of any efficient therapy known for the treatment of COVID-2019 infections and, also, to the process of developing new drugs is time-consuming and cumbersome, the use of bioinformatics as a tool can redirect old drugs against COVID-19, helping to identify treatments with known pharmacokinetic, pharmacodynamic and toxicity profiles [5]. Some recent studies have provided critical insights using bioinformatics and molecular modeling to help the rapid development of treatments that can be tested in clinical trials [16,17]. For example, using XML-like Web Effort (Q-UEL) systems to access relevant and emerging literature and interact with standard publicly available bioinformatics tools on the Internet helped quickly identify sequences of amino acids that are well conserved across many coronaviruses, including SARS-CoV-2. The theory behind Q-UEL has been described and developed in several essentially mathematical papers [17]. Q-UEL is a biomedical and pharmaceutical data mining tool that comprises knowledge bearing tags as “probabilistic statements” from relatively structured data sources [18] and specialist text [19], as well as “common sense” and general wisdom from thesauruses and encyclopedias, and automatic surfing of the Internet [20]. Research using this type of tool can contribute to the proposition of specific synthetic vaccine epitope and peptidomimetic agents (see [17]).

The role of bioinformatics in conjunction with molecular modeling in the search for methods of diagnosis, treatment and prevention of COVID-19 is unquestionable. Processes such as screening of bioactive compounds, modeling of biomacromolecule structures, primer selection and genetic sequencing compounds can be faster, more accurate and less expensive when aided by computer tools. Experts from all over the world believe in the potential of bioinformatics in combating the COVID-19 pandemic. Bioinformatics contributes to understanding the variations in SARS-CoV-2 proteins and how the virulence of this pathogen can increase. Based on this, this tool can also clarify how the virus subverts the immune system. Our know-how about the molecular elements involved in triggering this type of response can bring to light essential points that may otherwise be neglected.

In addition to issues related to bioinformatics and molecular modeling, understanding the participation of innate immunity in the infectious process is crucial for the screening of new target molecules for the treatment and diagnosis of COVID-19. It is well established in the current literature that innate immunity plays a central role in determining the outcome of viral infections in general [21]. Therefore, the present review explicitly focuses on this aspect of the immune system, demonstrating its role in the formation of the adaptive immune response downstream and its importance as a therapeutic target for the development of new drugs for the treatment of patients with severe COVID-19.

Researchers put much effort to understand the origin and pathophysiology of this novel coronavirus and have been testing multiple drugs to screen for therapeutic effective substances [22]. Despite little understanding about the pathophysiology and high pathogenicity of SARS-CoV-2 infection, early studies have shown that increased amounts of proinflammatory cytokines in serum (e.g., (interleukin IL) IL-1β, IL-6, IL-12, interferon-γ (IFNγ), Interferon-inducible protein 10 (IP10), and monocytic chemotactic protein 1 (MCP1)) were associated with pulmonary inflammation and extensive lung damage in patients with Severe Acute Respiratory Syndrome (SARS) [23]. Besides, research has shown that infection by MERS-CoV leads to a significant increase in serum levels of pro-inflammatory cytokines (IFNγ, TNF-α, IL15, and IL17) [24]. Patients who required Intensive Care Unit (ICU) admission had higher concentrations of GCSF, IP10, MCP1, MIP1A, and TNF-α than those who did not require ICU admission, suggesting that massive cytokine synthesis and secretion are associated with the severity of the disease [6].

Given the above, the present study aimed to develop a comprehensive review of aspects of bioinformatics and molecular modeling as auxiliary tools to pharmacology and immunology in the discovery of potential anti-SARS-CoV-2 agents. Understanding these aspects streamlines the biomedical research process, without any operational costs. Besides, we researched and gathered evidence of the interactions between SARS-CoV-2 and innate immunity. Discovering new therapeutic targets and understanding pathophysiological processes are the key to the development of effective treatments and efficient management of patients who progress to severe SARS-CoV-2 infection, which can save lives worldwide.

## 2. Coronavirus

Most coronavirus (CoV) infections in humans are caused by low-pathogenicity species, causing common cold symptoms; however, they can eventually lead to serious infections in higher risk groups such as the elderly, children, patients with comorbidities (hypertension, diabetes mellitus, asthma, among others) and/or those suffering immunosuppression. Prior to 2019, two highly pathogenic and animal-derived coronavirus species (SARS and MERS) were responsible for outbreaks of severe acute respiratory syndromes. Regarding human infection by SARS-CoV-2, the clinical spectrum is not fully described, and the lethality, mortality, infectivity, and transmissibility pattern is not yet fully elucidated. In addition, there is no specific treatment with antivirals that are effective against SARS-CoV-2 or available vaccines and, currently, treatment is supportive and nonspecific for COVID-19 in hospitalized patients with fever, accompanied by cough or sore throat and with dyspnea or O_2_ saturation below 95% or respiratory discomfort [25].

### Etiological Agent

CoV, of the order *Nidovirales*, *Coronavirin* subfamily and *Coronaviridae* family, is a single-stranded RNA virus with diameter of 80–120 nm [26] with appearance of crown under electron microscope (“coronam” is the Latin term for “crown”) due to the presence of glycoproteins in the viral envelope [27]. It is a virus capable of infecting humans and a wide variety of other mammalian hosts (e.g., mice, swine, rats, dogs, cats, rabbits, horses, cattle, cetaceans and bats) and birds (chickens, pheasants and turkeys) and develop respiratory, enteric, liver and central nervous system (CNS) diseases. Based on its genotypic and serological characteristics, CoV is classified into 3 subfamilies, previously called groups 1, 2 and 3. Group 1 and 2 were composed of CoV that has mammals as hosts and group 3 was composed, until recently, only of avian CoV [28,29,30,31,32]. Currently, the Study Group of the International Committee for Viral Taxonomy (ICVT) has proposed replacing the 3 traditional groups by subfamilies Alfacoronavirus (α-CoV) (group 1), Betacoronavirus (β-CoV) (group 2) and Gamacoronavirus (γ-CoV) (group 3). After that, the presence of a fourth CoV subfamily was detected in birds and pigs and called Deltacoronavirus (δ-CoV) [33,34].

The most common human CoVs (HCov) are HCoV-OC43, HCoV-HKU1, both β-COVs of strain A, and HCoV-229E and HCoV-NL63, both α- CoVs. Generally, they cause common colds and self-limited upper respiratory infections in immunocompetent individuals, that is, they are eliminated in a short period of time by the immune system without the need for intervention through specific pharmacotherapy. In immunocompromised and elderly individuals, lower respiratory tract infections may also occur. Other HCov include SARS-CoV, SARS-CoV-2 (or SARS-CoV-2) and MERS-CoV (β-COVs of lineage B and C, respectively). These CoV categories can cause epidemics of varying clinical severity, with respiratory and extra-respiratory manifestations. Regarding SARS-CoV, MERS-CoV, mortality rates are up to 10% and 35%, respectively [27] and SARS-CoV-2 belongs to β-CoV subfamily.

An important feature of the SARS-CoV epidemic between 2002 and 2003 was the virus efficiency in transmitting from species such as masked palm civet (*Paguma larvata*), raccoon dog (*Nyctereutes procyonoides*) and the Chinese ferret-badger (*Melogale moschata*) and infect human populations (fig 1a) [35].

**Figure 2:**
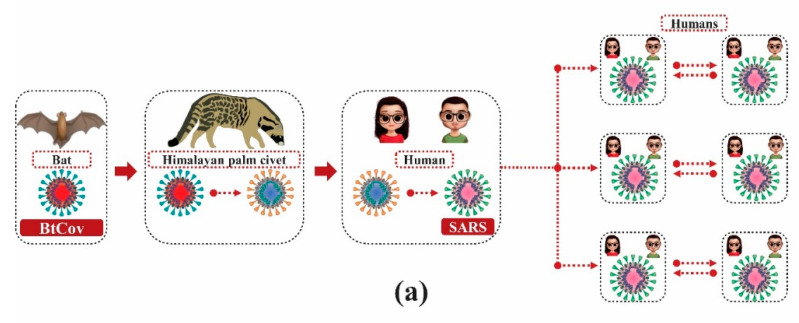

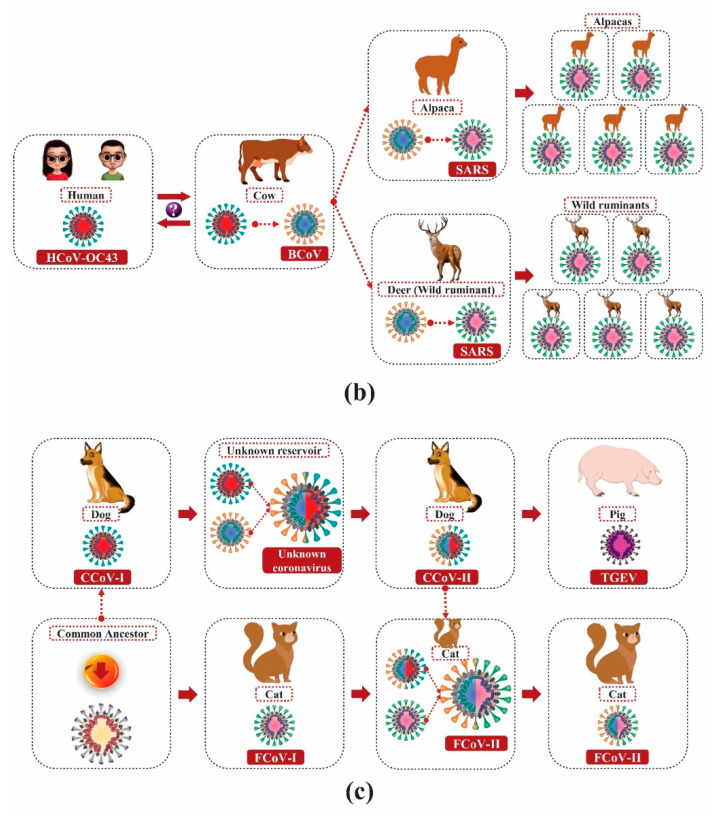
Transmission of coronavirus between different species: (**a**) Coronavirus whose natural reservoir are bats (BtCoV) is like coronavirus of the Severe Acute Respiratory Syndrome (SARS). This virus has spread and adapted to wild animals, for example, masked palm civet, which is marketed for human consumption in wholesale seafood markets in China. The employees of these markets that manipulate these wild animals have been infected; however, they did not present important clinical signs, and symptoms were minimal. The process of adapting the virus to new hosts resulted in strains with efficient replication capacity in human hosts, which cause diseases with clinical conditions ranging from mild to severe and with great ability to spread from person to person; (**b**) OC43 coronavirus, whose natural reservoir are humans (HCoV-OC43) and bovine coronavirus (BCoV) are closely related. It is postulated that these coronaviruses originated in another animal species and subsequently have crossed their species. BCoV has effectively spread among other animal species, for example, alpaca (South American mammal of the camelid family) and wild ruminants (such as deer); (**c**) Currently, some canine viruses are believed to have common ancestors with feline species. This occurs with coronaviruses that infect these species. Currently, feline coronavirus I (FCoV-I) and canine coronavirus I (CCoV-I) are believed to share a common ancestor. A recombination process (random exchange of genetic material) of CCoV-I with an unknown coronavirus gave rise to a second type of canine coronavirus (CCoV-II). The recombination of CCoV-II with FCoV-I in an unknown host gave rise to a second type of feline coronavirus (FCoV-II). There is evidence that CCoV-II was transmitted to pigs, originating the transmissible gastroenteritis virus (TGEV) [36]. Note: This image was developed using the CorelDraw software (2017 Corel Corporation ID 410003).

It is postulated that SARS-CoV is the result of the recombination of CoVs transmitted from these animals to humans. However, research has shown that SARS-CoV has not been detected in domestic or wild masked palm civet [36], suggesting that these, and other animals marketed in wholesale seafood markets in China, were not the main reservoirs of the virus [37]. CoV similar to SARS-CoV was isolated from the Chinese horseshoe bat (*Rhinolophus* spp.) [38,39], which were also marketed in live-animal markets, strongly suggesting that the virus may have recently been transmitted from bats to other mammals, such as masked palm civets, and later to humans (Figure 2a) [37].

In addition to SARS-CoV, there are other situations of cross-transmission of CoVs among different animal species. Bovine coronavirus (BCoV) and HCoV-OC43 have broad similarities. BCoV is believed to have been transmitted from bovine hosts to humans a hundred years ago [40]. Previous studies have demonstrated the isolation of BCoV from alpaca that showed signs of enteritis and in captive wild ruminants [41,42], strongly suggesting that this CoV has been transmitted to others species (Figure 2b).

Previous studies have shown that canine (CCoV), feline (FCoV) and swine coronaviruses exchanged genetic material at random, showing that they were infecting the same host. Recombination processes among the first CCoV and FCoV (CCoV-I and FCoV-I) strains and an unknown CoV resulted in two new groups of viruses, CCoV-II and FCoV-II. In addition, studies have shown that the sequence of the transmissible gastroenteritis virus (TGEV) genetic material shows that this CoV was originated through cross-transmission of CCoV-II species from an infected dog (Figure 2c) [43].

In general, statistical data suggest that 2% of the population are asymptomatic CoV carriers and that these viruses are responsible for about 5% to 10% of acute respiratory infections [44].

As for transmission and infection, rarely animal CoVs infect people and spread among them, as occurred with SARS-CoV and MERS-CoV. At first, many of the patients with outbreaks of respiratory diseases caused by SARS-CoV-2 in Wuhan, China, had some connection with a large seafood and live-animal market, strongly suggesting that the spread occurred from animal to people. However, after the outbreak began, an increasing number of patients supposedly did not have exposure to the animal market, also indicating the occurrence of spread from person to person [25]. The possibility of SARS-CoV-2 transmission from seafood to humans is unlikely. As the Wuhan seafood wholesale market also sells other animals, the SARS-CoV-2 natural host remains unknown [44].

The sustained spread from people to people is occurring in the globe. This culminated in the statement that the now-pandemic SARS-CoV-2 outbreak constitutes a Public Health Emergency of International Concern (PHEIC) by WHO on January 30, 2020 in Geneva, Switzerland. Person-to-person transmission cases have already been reported worldwide. Transmission in health institutions, such as hospitals, has frequently occurred, having already been reported in almost all countries where SARS-CoV-2 is present.

Regarding the person-to-person spread that occurred with SARS-CoV or MERS-CoV, the main means is believed to have been through respiratory droplets produced when an infected person coughs or sneezes, similar to the way influenza and other respiratory pathogens are disseminated. In addition, aerosol transmission has been identified in patients undergoing airway procedures, such as endotracheal intubation or airway aspiration. In the population, SARS-CoV and MERS-CoV dissemination among people usually occurs after close contacts, and health professionals involved in assisting these patients are particularly vulnerable [25].

A recent study conducted with women who were in the third trimester of pregnancy and who were confirmed with SARS-CoV-2 infection showed no evidence of vertical transmission, that is, from mother to child. Despite that, all pregnant women underwent cesarean section and it remains unclear whether transmission can occur during normal delivery [45]. Further studies with pregnant women in other gestation periods and even in the third trimester should be carried out, since the sample used was small. Studies like this are crucial for the care of pregnant women, since they are relatively more susceptible to respiratory pathogen infection and severe pneumonia.

Recent studies have shown that interpersonal SARS-CoV-2 transmission has occurred since mid-December 2019 in Wuhan (China) and from there, considerable efforts to reduce transmission and control the current pandemic will be necessary, since the transmission dynamics is very similar in all places where the number of infected and dead people is high. Furthermore, these results suggest that measures to prevent or reduce transmission should be urgently implemented in populations at risk [1]. For this reason, all government spheres worldwide, together with regulatory agencies from the health area, recommended complete social distancing as an emergency measure to contain the spread of SARS-CoV-2 through interpersonal contact. It is important to highlight that, through the experience of Chinese and Italians, the possibility of SARS-CoV-2 transmission before the onset of symptoms cannot be excluded and although it seems uncommon, there is evidence that individuals who remain asymptomatic can transmit the virus. These data suggest that the use of isolation is the best way to contain the SARS-CoV-2-associated pandemic [27].

China’s CDC and local CDCs used the Chinese experience in the SARS-CoV-2 outbreak to conduct investigations that determined the viral incubation time. They demonstrated that this period can vary from 3 to 7 days to 2 weeks, since the longest time from infection to symptoms was 12.5 days (95% CI, 9.2–18) [1]. In addition, these results showed that the new pandemic doubled every seven days, while the basic reproduction number (R0—R zero) is 2.2, that is, each patient transmits the infection to an additional 2.2 individuals, on average. It is important to note that the R0 estimates of the SARS-CoV epidemic in 2002–2003 were approximately 3 [46].

For viral infection to occur, the connection of the virus to a receptor expressed by host cells is the first step, followed by fusion with the cell membrane. Specifically for SARS-CoV-2, it is possible to deduce that the epithelial cells of lungs are its main target, since in part of infected patients, COVID-19 progresses as an acute respiratory infection [47].

**Figure 3:**
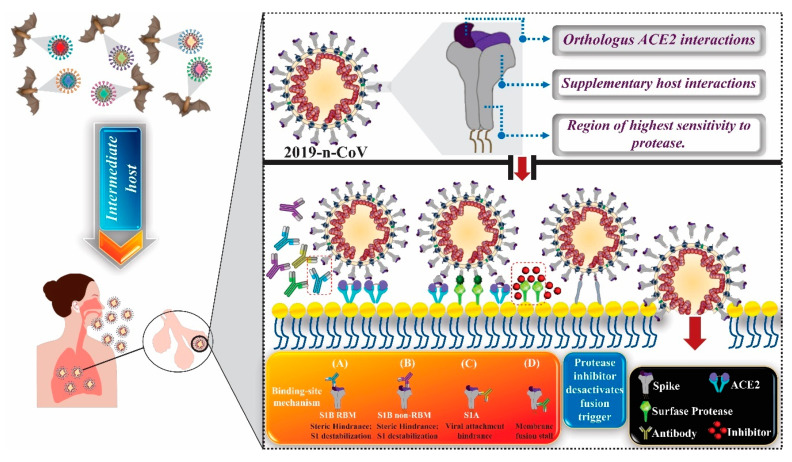
Infection of pneumococytes during COVID-19. It is assumed that CoVs that cause COVID-19 in humans are related to bats (upper left corner). This selected subset of viruses has the necessary resources to infect the human respiratory tract (lower left corner), with a certain tropism for this system. Infection (right panel) requires the interaction of spike proteins present in the SARS-CoV-2 viral envelope (S proteins) with host sites for type 2 angiotensin-converting enzyme (ACE2) present in the lung. Subsequently, proteases present on the surface of pneumocytes cleave the S2 region of the S protein, the subunit responsible for the fusion of the S protein with the cell membrane. After cleavage, a series of conformational changes are triggered, resulting in the fusion between viral envelope and the target cell membrane. The structural features of SARS-CoV-2 that can facilitate infection in humans include: (1) Presence of reasons for binding to the S1B receptor (RBMs) (in purple) that bind to ACE2 orthologous receptors. ACE2 is believed to be orthologous because it exhibits homology to S1B RBMs (since they complement each other to the point of binding) and were probably duplicated from a common ancestor, shared by the two underlying sister species, where in the course of evolution, both receptors gradually differentiate but continue to have affinity for each other; (2) An S1A domain that provides additional interactions with the host and; (3) A cleavage substrate for a furin protease (represented by the green starry shape bound to the protease at the bottom right of the figure), which can provide greater sensitivity to cleavages by host proteases. Anti-CoV antibodies (shown at the bottom right of the figure) can prevent infection through the following mechanisms: (A) binding to S1BRBMs of the virus, blocking access to ACE2 receptor and consequently preventing the continuation of the process of virus fusion with the target cell; (B) distal connection in relation to RBMs, generating steric impairment and, consequently, blocking the connection between the virus RBMs and the ACE2 receptor of the host cell; (C) binding in the S1A region of the viral spike, blocking alternative connections to different receptors; and (D) binding to S2, the region responsible for the fusion of the virus with the membrane of the target cell, consequently preventing fusion. As future perspectives, future research should aim at the development of protease inhibitor antiviral compounds, which play a crucial role in the fusion of the virus to the host cell membrane, suppressing the entry of the virus [50]. Note: This image was developed using the CorelDraw software (2017 Corel Corporation ID 410003).

Previous studies have shown that human-to-human SARS-CoV transmission occurs by the association between the binding domain to the spike receptor located on the viral envelope and the cell receptor. Recently, it was identified that cell receptor for SARS-CoV is the angiotensin-converting enzyme 2 (ACE2) [48,49]. It is important to note that the sequence of the binding domain to the spike receptor of the SARS-CoV-2 viral envelope is similar to that of SARS-CoV. These data strongly suggest that the entry into host cells occurs through the ACE2 receptor (Figure 3) [48].

In a recent study, [7] demonstrated the interactions of SARS-CoV-2 with the host organism, specifically interactions between spike proteins (“coronan”) located in the viral envelope (S proteins), projected from the envelope, and the host ACE2 receptors. CoVs derived from the most virulent bats are those in which S proteins have tropism by distinctly human receptors, as is the case with SARS-CoV-2. After the first contact with humans, the virus migrates to lungs and S proteins interact with various proteins and receptors, which are factors of host susceptibility. Such interactions cause substantial changes in the conformation of proteins involved in the infection process of host cells, triggering the fusion between virus and human cells and, consequently, the infection. It is noteworthy that specific antibodies against S proteins and antiviral agents that block the link between virus and human susceptibility factors can prevent SARS-CoV-2 infection [51] (Figure 2).

Studies like these have shown that there is a wide spread of CoVs in their natural reservoirs and by demonstrating interactions between virus and human hosts and, consequently, how the infectious process occurs, they drive the development of vaccines and antiviral drugs. In addition, further studies should be aimed at the active surveillance of these viruses in geographic regions that go beyond the beginning of the SARS-CoV-2 pandemic outbreak. In the long term, broad-spectrum antiviral drugs and vaccines should be developed with the aim of controlling emerging infectious diseases caused by CoVs. In addition, regulatory agencies in the health sector should develop specific regulations to control domestication and consumption of wild animals.

## 3. Innate Immunity and Coronavirus

The emergence of the highly pathogenic SARS-CoV-2 led to the need for deeper knowledge on the biology and pathogenesis of CoV. An emerging theme in the CoV pathogenesis and COVID-19 pathophysiology is the interaction between specific viral genes and the host’s immune system, which acts as a key determinant in the regulation of disease virulence and development results.

Viral interactions with innate immune system play a key role in determining the course of the infection. The initial control of viral replication by type I interferons (IFN), complement system proteins and other innate immune mediators limit viral spread in the host during the early stages of the disease [52]. In addition, the initial innate response also plays a key role in the development of the subsequent adaptive immune response [53]. Despite that, it is well established in current literature that the hyperactive innate immune response can also result in pathology and subsequent tissue damage.

**Figure 4:**
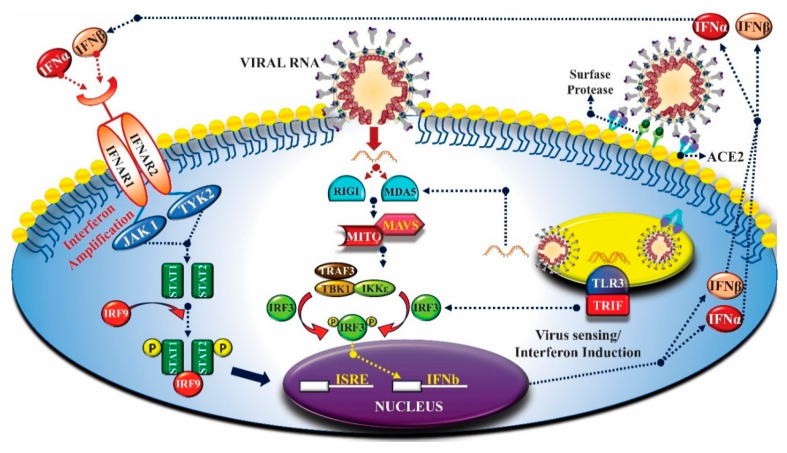
IFN signaling and synthesis pathway. RNA viruses can be internalized in the target cell by different mechanisms: (A) fusion with the plasma membrane or; (B) binding to a surface receiver (ACE2 for SARS-CoV-2 and SARS-CoV). After internalized, the virus exposes the genomic RNA to the dsRNA detection mechanism in the cell, that is, TLR3, RIGI and MDA5. These proteins are responsible for the IRF-3 cascade signaling, leading to IFNb induction and, consequently, the production of IFNβ protein. The newly synthesized IFNβ can bind to IFN receptors on the surface of the same cell or surrounding cells and induce the synthesis of more IFN molecules. Binding to IFN receptors activates the signal transducer and activator of transcription 1 (STAT1) signaling pathway to activate several distinct antiviral genes located in ISRE promoter elements [21,55]. Note: This image was developed using the CorelDraw software (2017 Corel Corporation ID 410003).

Regarding innate immunity, there are two main pathways by which cells detect viruses that invade the organism and activate the IFN pathway [21]. Toll-like receptors (TLRs), which include TLR3, TLR7, TLR8 and TLR9, are responsible for detecting viruses in endosomal compartments as they penetrate host cells. The cytoplasmic domain of TLRs (called CARD) contains two specific RNA-helicases, RIG-I and MDA5, which detect viral RNA in the cell cytoplasm. For activation of both pathways, interactions between sensors with pathogen-associated molecular patterns (PAMPs) are required, such as, for example, single-stranded RNAs associated with viral genomes or double-stranded RNA, which is a by-product of viral replication, being common targets. Different adapter proteins participate in signal transduction in the induction pathways of TLRs and cytoplasmic IFN. The TLR-dependent pathway uses TIR-domain-containing adapter-inducing interferon-beta adapter proteins (TRIF) and/or the myeloid differentiation factor 88 (MyD88) and the IFN cytoplasmic induction pathway uses the MAVS/IPS-1/VISA/CARDIF mitochondrial adapter protein [54] (Figure 4).

The complement system plays a crucial role in the innate immune response to viruses. It represents one of the main factors responsible for the development of pro-inflammatory reactions during these diseases [56,57]. Research has shown that, just as in human infection, intranasal SARS-CoV infection in C57BL/6J mice, results in virus replication with high rates in the lung, in addition to induction of inflammatory cytokines and chemokines and infiltration of immune cells in the pneumocytes [58]. Using C3-deficient mice (C3 ^-/-^), these researchers demonstrated that animals C3 ^-/-^ infected with SARS-CoV lost less weight and obtained a significant reduction in respiratory dysfunction when compared to control groups, despite viral loads equivalent in the lung. Besides, a significantly lower rate of neutrophils and inflammatory monocytes were present in the lungs of C3 ^-/-^ mice than in the C56BL/6J controls, and subsequent studies showed that lung injury was reduced, as were cytokine (IL-6, mainly) and chemokine levels in lungs and serum of C3 ^-/-^ mice than when purchased from animals in the control group [58]. This suggests that C3 inhibition may significantly reduce the pulmonary inflammatory complications of SARS-CoV-2 infection. The decrease in neutrophilic infiltration in the lungs and the reduced levels of intrapulmonary and plasma IL-6 that have been observed in mice with C3 ^-/-^ infected with SARS-CoV suggest a potential treatment that combines C3 inhibitors and anti-IL-6 drugs [59]. Besides, as C3 is one of the first proteins synthesized by the complement system in the innate immune cascade, the anti-inflammatory potential for C3 blockage is broader. Drugs like AMY-101 are being investigated for this [60], which is currently being tested in patients with COVID-19 [59]. C3 blockade can simultaneously inhibit the synthesis of C3a and C5a. Furthermore, this block reduces the intrapulmonary activation of C3 and the release of IL-6 from alveolar macrophages or other cells that express C3a receptors (C3aRs) and/or C5a receptors (C5aRs), causing the beneficial evolution and cure of lung injury [60]. 

The role of complement system activation in the development of severe acute respiratory syndrome associated with SARS-CoV-2 infection is not yet fully understood, and clinical data are scarce. Recent research has shown that the SARS-CoV, MERS-CoV, and SARS-CoV-2 “N” proteins bind to MASP-2, the essential serine protease in the complement activation lectin pathway, leading to over complement activation and lung injury severe inflammatory disease. Thus, it is evident that the blockade between the interaction of protein N with MASP-2 can significantly reduce the hyperactivation of complement induced by protein N and lung injury in vitro and in vivo.

**Figure 5:**
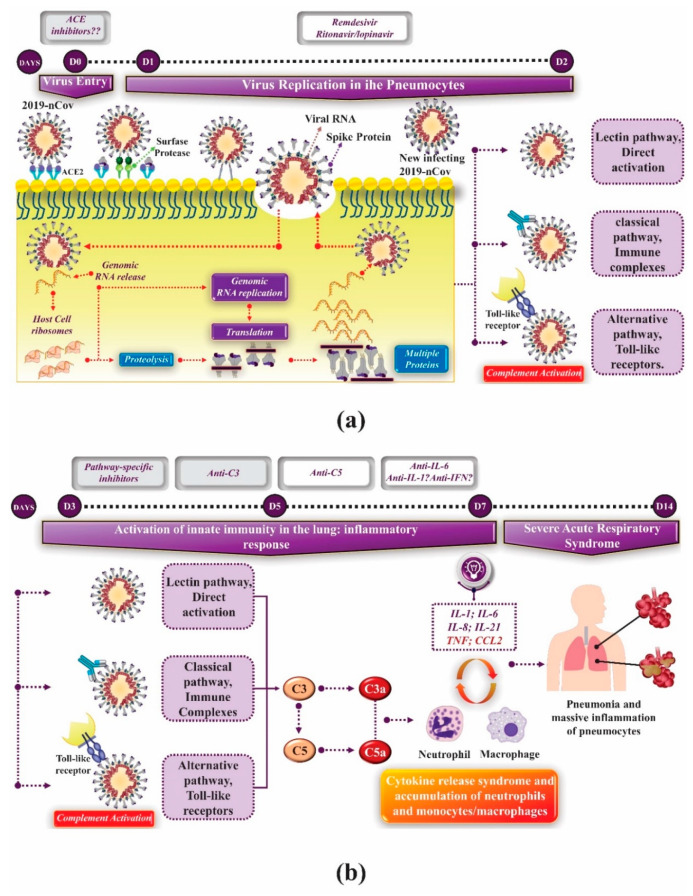
A possible scheme for using complement system inhibitors in lung injury associated with SARS-CoV-2: (**a**) SARS-CoV-2 penetrates the host’s pneumocytes and uses the cellular machinery for protein synthesis and replication of the genetic material and causing activation of the complement system through different pathways; (**b**) Complement activation contributes to the massive inflammatory response of pneumocytes observed in some patients with severe COVID-19. The inhibition of C3 or C5 can have significant therapeutic potential [59]. Note: This image was developed using the CorelDraw software (2017 Corel Corporation ID 410003).

Complement overactivation is present in patients with COVID-19, and a promising suppressive effect has been seen in patients with severe lung injury treated with an anti-C5a monoclonal antibody [61]. Due to the cascade organization of the complement system, inhibitors that target C3 or its upstream activators may be more effective and, potentially, may prevent the initial stages of the infectious process that lead to severe lung inflammation. Despite this, no substance that acts with these mechanisms of action has been approved for use in humans, although phase II clinical studies are already underway [59]. Figure 5 outlines the moment when complement system inhibitors can be used in lung injury associated with SARS-CoV-2.

## 4. Adaptative Immunity and Coronavirus

The mechanism of natural SARS-CoV-2 infection occurs very similar to previously studied SARS-CoV [62] and produces adaptive immune responses against the structural antigens of these viruses in humans and animals [63]. Spike glycoproteins located in the viral envelope (S proteins) are responsible for binding to the cell receptor and fusing the virus membrane with the host’s cell membrane [64]. These glycoproteins also act as antigens responsible for the activation of T cells and development of humoral and cellular immunity [65]. Literature data have demonstrated that the majority of antigenic peptides are located in structural proteins (mainly S protein) [66,67].

It is believed that hypervirulent CoV variants, such as SARS-CoV-2, have the capacity to develop viremia, producing systemic disease, being often fatal [6,68,69,70,71,72,73]. Previous studies have shown that SARS-CoV infection of macrophages and dendritic cells leads to an aberrant cytokine/chemokine expression pattern [21], so that the ability to infect and replicate in such phagocytes appears to be a determinant factor for establishing the course of viral infection. In addition, it has recently been reported that SARS-CoV-2 causes lymphocyte depletion, resulting in high viral titers [6,37,74,75].

Previous studies demonstrate the spread of SARS-CoV to lymphoid organs such as spleen, thymus, Peyer plaques and mesenteric lymph nodes, developing intense lymphoid depletion [69,73,76]. It is already well established in literature that lymphocytes and their subtypes are essential to maintain the immune system function in basal activity or during infectious processes. Viral infections, immunodeficiency syndromes and other infectious diseases, such as tuberculosis, are known to determine abnormal plasma counts in lymphocyte levels and their subtypes [77,78,79]; and for such infections to evolve into more serious events, lymphopenia is crucial, since the reduction of this cell type is necessary for the survival and persistence of many microorganisms. In Wuhan, where SARS-CoV-2 infection started, 63% of patients with COVID-19 had significant lymphopenia induction [6].

Although the mechanisms that cause lymphopenia in CoV infections are not fully understood, it is already known that lymphocytes can undergo lysis by direct or indirect viral action [70]. Previous studies have shown that indirect events, secondary to viral infection, are the main responsible for cell lysis through the synthesis of soluble factors such as IFN, cytokines and chemokines by host cells [70,73].

As already mentioned, very recent studies have identified that the SARS-CoV cell receptor is ACE2 and that the sequence of the binding domain to the spike receptor of the SARS-CoV-2 viral envelope is similar to that of SARS-CoV, suggesting that the entry into host cells occurs through the ACE2 receptor [48]. However, the presence of ACE2 receptors has been identified in the oral mucosa, in type II pneumocytes, along the intestine and in the renal and cardiac endotheliums [80], with no evidence of the presence of these receptors in circulating mature lymphocytes. For this reason, it is believed that the cytolytic effect of SARS-CoV-2 that led infected patients to develop lymphopenia is not direct, that is, caused by the virus itself, since the receptor determines the tropism of the pathogen by the tissue. In addition, previous studies have demonstrated the presence of COV RNA in lymphoid tissues, such as the thymus, in infected patients. This suggests that one of the lymphopenia mechanisms in these patients may be related to the reduction in the production of mature lymphocytes by the thymus, which is the target organ in several infectious diseases, including coronavirus [81]. Since these viruses infect the thymus and cause tissue damage, there is an important interference in the lymphocyte differentiation process, which can cause lymphopenia [82].

It is well established in literature that T cells are crucial to produce immunoglobulins and, consequently, for the development of humoral and memory immunity. Therefore, pharmacological treatments or the use of integrative and complementary practices that prevent the reduction or increase the levels of cells of the immune system can be essential tools in combination with treatment and/or prevention of SARS-CoV-2 infection.

## 5. Use of Bioinformatics and Molecular Modeling as Technology Applied to Health to Diagnose and Treat COVID-19

The use of bioinformatics and other computational tools in addition to molecular modeling has helped researchers from different areas in the search for strategies for diagnosing viral infection, in the development of vaccines for its prevention, as well as in the discovery of new anti-SARS-CoV-2 agents.

The knowledge of the genome of a species based on the genetic sequencing technique is the starting point for the structure and function of its genes to be understood. In the context of diseases transmitted by microorganisms, such as SARS-CoV-2, the mapping of the genome of microorganisms collected from infected patients in different regions of the world also allows tracing a transmission profile, including its dissemination in different regions and countries, contributing to the search for strategies to combat the disease and monitor mutations [16,83]. Data from the NCBI GenBank^®^ gene sequence database https://www.ncbi.nlm.nih.gov/genbank) accessed on 08/04/2020 indicated more than 14,000 nucleotide sequences inserted since December 2019 for SARS-CoV-2, most of them coming from cities in China and the USA. In February 2020, researchers from the University of São Paulo and Instituto Adolf Lutz, in Brazil, together with researchers from the University of Oxford, in the United Kingdom, managed to elucidate and publish the complete gene sequence of the virus (GenBank accession number MT126808) obtained from of the first confirmed cases of the disease in Brazil within just two days from the confirmation of the diagnosis [84]. The speed in obtaining this information is only possible through the application of bioinformatics techniques.

**Figure 6:**
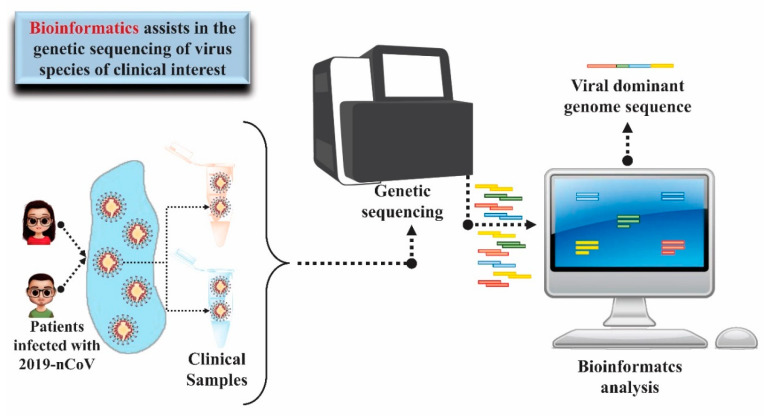
Bioinformatics as technologies applied to health as allies to coping with the disease: Bioinformatics is a technology that assists researchers in coping with diseases by investigating genetic sequencing and seeking structural models of potential molecular targets present in SARS-CoV-2. Note: This image was developed using the CorelDraw software (2017 Corel Corporation ID 410003).

Bioinformatics showed great advance in the 1990s, mainly in view of the development of the genomics area, which generated large amount of biological data incompatible to be quickly analyzed in a manual way. The rapid and adequate manipulation of these data has been possible through the application of data comparison and analysis software and easy access to previously available data from the sharing and storage of information in virtual databases [85]. In view of experimentally obtained sequences, it is possible to perform the alignment of these sequences to other sequences available in virtual databases, leading to the knowledge of the family to which the microorganism is related [83,86,87]. In one of the first studies on the new virus that caused respiratory infections in China, the researchers were able to determine from the sequencing of RNA obtained from bronchoalveolar fluid samples that the etiologic agent was RNA virus of the Coronaviridae family. In addition, using bioinformatics tools, it was possible to perform a phylogenetic analysis, revealing 89.1% similarity of the nucleotide sequence of this virus with a group of coronaviruses of the genus Betacoronavirus already identified in bats in China, which gave evidence of the virus origin [88] (Figure 6).

One of the algorithms most widely used for comparing biological sequences, whether from nucleotides to nucleic acids or from amino acids to proteins, is the BLAST (Basic Local Alignment Search Tool) [89]. The diversity of information on biological sequences from organisms of different degrees of complexity allows BLAST to be a starting point to understand the genetic relationship with other species, also tracing a possible origin. The use of such tool allowed [90] to identify high similarity between SARS-CoV-2 genomic sequence extracted from NCBI GenBank^®^ (GenBank accession number MN908947) and viral metagenomas found in the pangolin mammal present in the database. These studies, in turn, guided analyses that led to findings of high amino acid identity of S, E, M and N genes of pangolin coronavirus with those isolated from humans, suggesting that the virus capable of infecting humans may have emerged from a recombination of coronaviruses isolated from bat and pangolin.

The large capacity for storing information in virtual databases made possible by advances in the field of information technology allows these databases to be continuously updated with new genomic sequences to be universally available. In the context of COVID-19, this characteristic was important for a better understanding of the origin of SARS-CoV-2 from the comparative analysis of genomic data of the new virus with others from the same family, suggesting its origin from natural selection, with modifications in its spike protein, more specifically in the host receptor binding domain, which may have enhanced its interaction and recognition by the human cell [83,91].

The RT-PCR test is considered the gold standard for diagnosing COVID-19 [92]. By this technique, the reverse transcriptase enzyme initially participates in transforming the viral RNA into complementary DNA (cDNA). Then, the genetic material is amplified. In this process, the regions of the genetic material to be amplified are recognized by polymerase by means of oligonucleotides complementary to the region of interest called primers [93]. The step of primer selection is essential to ensure the quality of the analysis and requires a thorough analysis of the reference genetic sequence [94]. It is also important to consider that recognition is specific, and the method can be applied worldwide, that is, genetic material conserved regions should be prioritized over regions of low similarity. In this context, bioinformatics can contribute by allowing the design of primers based on the comparison of different genetic sequences from different geographic regions, available in virtual databases, identifying regions conserved in the genome, which would enhance the method’s specificity [95]. In the work of [92], which is also recommended by WHO as a reference in primers to be used in RT-PCR tests, researchers used complete and partial genetic sequences of SARS-related viruses present in the NCBI GenBank^®^ database for the design of primers and, in view of the first publications of SARS-CoV-2 sequence on Virological.org [84] and GISAID [96], it was possible to align the primers proposed for these sequences and to select those with greater correspondence. Several online servers and software have been used in order to optimize this process [95,97,98].

One of the main strategies to control viral diseases has been vaccination prevention. The development of a vaccine involves the identification of either synthetic or natural antigens that can prevent or even treat a disease. For viral diseases, the most explored strategies involve the use of attenuated viruses in their composition, use of viral structures that can be neutralized by the immune system, or application of part of the viral genetic material related to the expression of proteins that can behave as antigens for the development of antibodies against the pathogen of origin [99]. Regardless of strategy to be used, knowledge about the viral genome and the structure and function of virus proteins is essential for a vaccine to be successfully developed. In this context, bioinformatics tools have also contributed to greater quality and speed in the process. The proposal of vaccines based on B and T-cell epitopes has been a possible strategy for obtaining vaccines for COVID-19 prevention. Such epitopes can be predicted from the analysis of amino acid sequences of proteins of the SARS-CoV-2 pathogen and comparison with virtual epitope databases [100,101]. Even without studies on specific immune responses against SARS-CoV-2, the genetic similarity between this virus and SARS-CoV obtained from sequential alignment and phylogenetic analysis of genomes indicates that possible common epitopes related to structural S and N proteins could be incorporated into vaccines to be developed, in view of the immunological response already observed against SARS-CoV structures [102]. The search for regions conserved in genetic sequences of different coronavirus species aided by bioinformatics tools has also been a way to identify sequences of amino acids that can be models for the design of specific synthetic epitopes to compose vaccines for COVID-19 prevention [17].

The discovery and planning of new drugs that can be used in the treatment of COVID-19 benefited from the several advances observed over the last decades in the field of bioinformatics. A potential antiviral agent should target processes or macromolecules essential for maintaining the cycle of viral replication and infection of new host cells [103]. To do so, knowing the genetic sequence of the virus is not enough, but it is also important to determine the functions related to each of the genes that compose it. The sequential identity between genetic materials of different organisms may indicate equivalent genes in terms of function [104], which encode common proteins that can be exploited as molecular targets for the action of drugs aimed at the treatment of viral infections, for example [105]. Prior knowledge of potential molecular targets in other coronaviruses combined with the similarity of genetic sequences among viruses of this family opens several doors for the discovery of new bioactive compounds aided by computational tools [11].

To modulate the action of a molecular target, it is important to know its three-dimensional structure. The determination of this structure is essential for the understanding of how its connection site is organized, how it interferes with its mechanism of action and, given this information, how agents that interfere with its functioning can be proposed. Information on the structural organization of a protein is obtained by various techniques such as X-ray crystallography [106], cryogenic electron microscopy [107] and Nuclear Magnetic Resonance (NMR) [108]. However, bioinformatics tools are those that enable the detailed analysis of data obtained by these techniques and, consequently, the proposition and refinement of three-dimensional models, also exploring comparative analyses with models already proposed [109].

In the same way that there are virtual databases powered by genetic sequences, advances in bioinformatics have also enabled the creation of virtual databases of models of three-dimensional structures of biomacromolecules, which data are freely accessible and can be downloaded and viewed using visualization and molecular modeling software. The most popular three-dimensional structure database is the Protein Data Bank (PDB—www.rcsb.org), with more than 160,000 structures inserted so far [110]. So far, more than 300 structural models of SARS-CoV-2 macromolecules were deposited in the PDB. Most refer to structures proposed for the main protease complexed or not with possible inhibitors [11,111], but structural models can also be found for the nucleocapsid protein, non-structural proteins Nsp3, Nsp9, Nsp11, Nsp15 [112] and Nsp16 and spike glycoprotein [113,114].

The determination of structural models of the main SARS-CoV-2 protease, an important enzyme in the processing of polyproteins translated from viral RNA in free forms and complexed with the α-ketoamide 13b inhibitor [11] started from obtaining crystallographic data via X-ray diffraction. Structural models were then determined by the molecular substitution method, using as reference a structural model from SARS-CoV already available in PDB, with 96% sequential identity. Previously determined SARS-CoV spike glycoprotein models were also used as a reference in the construction of models of the same SARS-CoV-2 protein, whose structural data had been obtained from cryogenic electron microscopy [114].

The construction of structural models using crystallographic data as a reference allows the knowledge of the real arrangement of the structure under given experimental condition. When determining the structure of the main protease complexed with 13b inhibitor, [11] were able to “visualize” at molecular level, aided by computational tools, the main points of interaction between inhibitor and enzyme, which may contribute to direct structural modifications that lead to the optimization of the compound’s inhibitory activity. Likewise, the knowledge of interactions involved in the protein-protein complex between the RBD domain of the SARS-CoV-2 spike glycoprotein and the human cell ACE2 receptor helps to better understand how viral recognition occurs and the consequent search for epitopes conserved in this viral protein that can be targets of neutralizing antibodies to enable the production of vaccines [113].

Obtaining crystals from biomolecules; however, can be a laborious or even unfeasible process [115]. In these situations, we choose to build structural models using homology modeling. By this technique, a macromolecule of known three-dimensional structure with considerable degree of sequential identity to that to be determined is used as reference for the construction of this structural model [116]. Comparative studies between models determined with the aid of crystallography and models obtained by homology modeling indicate that they have high prediction quality and can be widely applied in studies of interaction between ligand and molecular target [117,118,119]. Considering the urgency of obtaining structural models of potential molecular targets present in SARS-CoV-2 and the availability of models of three-dimensional protein structure, mainly of other viruses of the Coronaviridae family, with sequential similarity to those of the virus that causes COVID-19, homology modeling is the fastest, cheapest and most accessible way for structural determination. Thus, many research groups have used this strategy to obtain structural models of different SARS-CoV-2 proteins, such as the nucleocapsid protein [120], envelope protein, ORF7a protein [88,120], non-structural proteins (Nsp) [88,120,121], 3CLpro [121,122] and spike glycoprotein [88,120,123], which have been used for the discovery of antiviral agents and the development of vaccines against COVID-19.

The use of molecular modeling at the early stages of searching for new bioactive compounds has contributed for the faster development of new drugs at reduced costs. In view of the urgency for effective treatments of COVID-19, virtual screening methods for bioactive compounds end up by optimizing this discovery pathway by allowing the initial selection of those that present steric and electronic similarities with compounds of known activity (drug planning based on the ligand) or that have the potential to strongly bind to the molecular target of interest (structure-based drug planning) [124]. In view of the low number of compounds with known anti-SARS-CoV-2 activity and considering the availability of structural models of potential molecular targets of the virus, the strategy based on the structure of the molecular target has been further explored, in which molecular docking techniques play a key role in this planning method.

Molecular docking consists of anchoring a molecule at the binding site of a molecular target, evaluating its assumed conformations, always searching for the one that generates the lowest-energy complex or the most stable with the target [125]. The binding energy of this complex can be related to its ability to interact and thus modulate the target’s action. Based on this information, it is possible to select, from virtual databases with a multitude of compounds, potential inhibitors, agonists, or antagonists, against a specific target, which will be hit compounds for more targeted structural changes.

**Figure 7:**
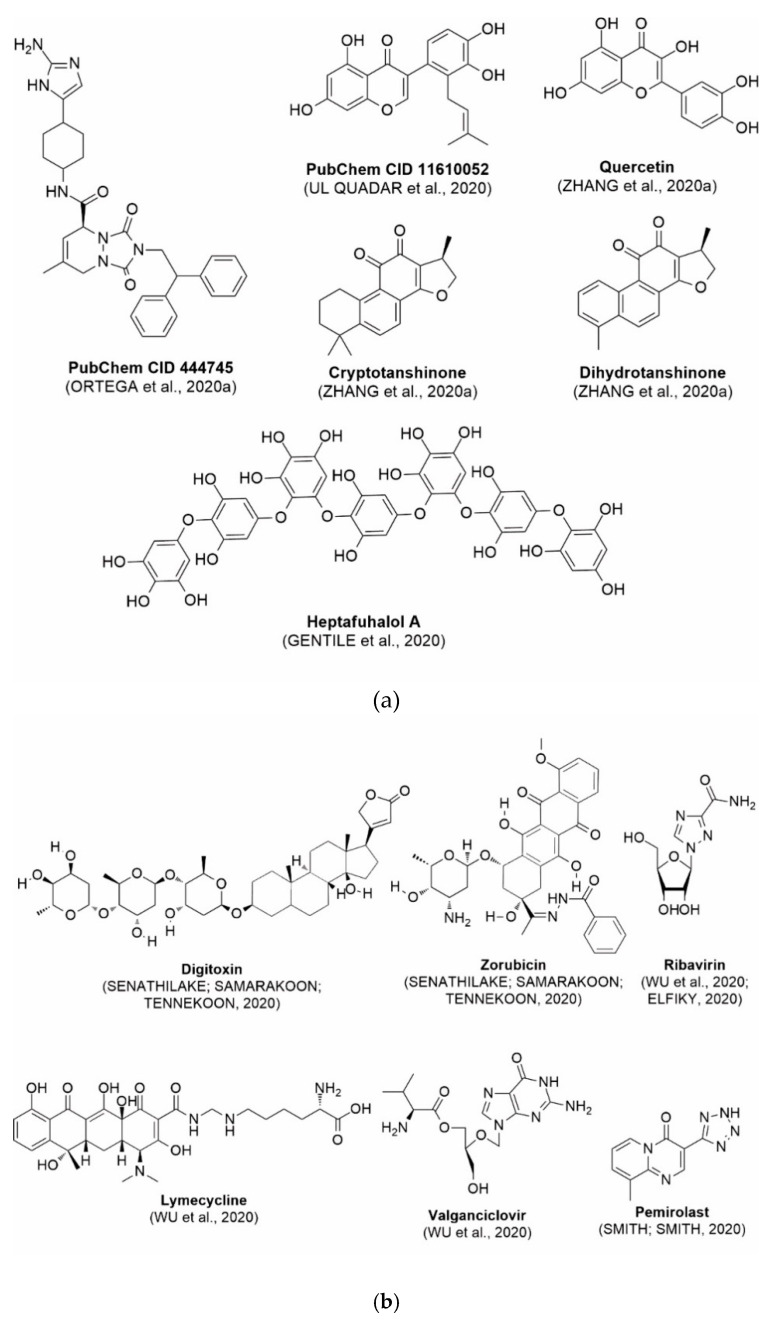
Structures selected from virtual libraries of chemical compounds via virtual screening: (**a**) Compounds obtained from chemical libraries with potential anti-SARS-CoV-2 action; (**b**) Drugs with potential for repositioning in the treatment of COVID-19.

In this process of identifying new hit compounds via virtual screening, virtual libraries of chemical compounds, such as ZINC databases (https://zinc.docking.org/) [126] and PubChem (https://pubchem.ncbi.nlm.nih.gov/) [127], offer a great diversity regarding structural patterns and origin (natural or synthetic), which can contribute to higher quality in the search process. These two databases were explored by [128] to identify possible inhibitors of the SARS-CoV-2 main protease. From these chemical libraries, researchers selected compounds that already had the ability to inhibit proteases found in other organisms, totaling more than 300 tested compounds, which were submitted to molecular docking studies against the main protease (PDB ID 6LU7), thus determining the interaction energy of the target-ligand complex. In this study, the PubChem CID 444,745 compound (Figure 7a) showed the greatest potential for inhibiting the enzyme, forming the most stable complex with the main protease.

There are published works that have focused on the screening of compounds of natural origin, which have great historical importance in the process of discovering new drugs [129]. Libraries of phytochemical compounds with antiviral action from traditional Chinese medicine plants have already been virtually screened against different SARS-CoV-2 molecular targets such as the 3CLpro protein [122,130], papain-like protease (PLpro) and spike glycoprotein [130]. Among selected compounds, PubChem CID 11,610,052 [122] and quercetin [130] showed strong interaction with 3CLpro; dihydrotanshinone I with the spike glycoprotein; and cryptotanshinone, with both PLpro and 3CLpro proteins [130] (Figure 7a). In addition to phytochemicals, compounds of marine origin have also been in silico evaluated against the SARS-CoV-2 main protease [131]. The virtual screening strategy adopted by the authors involved the initial construction of a pharmacophoric model based on the structure, which was created from information on the structure of the enzyme cocrystallized with N3 inhibitor (PDB ID 6LU7). In this work, the virtual screening of structures from libraries of marine compounds was then carried out using the pharmacophoric model created as a molecular filter to select those that presented structural characteristics corresponding to the created model. Subsequently, the selected compounds were submitted to anchoring at the active site of the enzyme as a way of assessing complementarity with the target. Different compounds were identified as potential main protease inhibitors in this analysis, highlighting the compound with the greatest potential heptafuhalol A (Figure 7a).

In addition to the virtual screening strategies adopted, another that has stood out among works searching for new anti-SARS-CoV-2 agents is the one that uses drug groups already approved, preferably those with antiviral action, such as search libraries, characterizing a drug repositioning strategy. The interest in studying compounds already in pharmaceutical use for other purposes has the advantages of reducing time and costs mainly related to the stage of drug discovery, but above all the fact of knowing the toxicological profile of the compound, previously attested in clinical trials [132]. In two studies found in literature, the use of a set of drugs obtained from the ZINC database [126] was observed as a library of screening compounds. In the work of [133], the interaction between this set of drugs and the SARS-CoV-2 spike glycoprotein was evaluated, highlighting as a result the high potential for interaction between digitoxin and zorubicin (Figure 7b). In the work of [88], the set of drugs obtained from ZINC was virtually evaluated regarding their interaction with different probable molecular targets of the virus, highlighting the potential of ribavirin against PLpro; limecycline against 3CLpro and valganciclovir against RNA-dependent RNA polymerase (RdRp) (Figure 7b). In the study by [134], the drugs analyzed were obtained from the SWEETLEAD database [135] and screened against the spike glycoprotein, with drug pemirolast (Figure 2b) being indicated as a promising inhibitor of the action of this glycoprotein. [136] conducted in silico evaluation against SARS-CoV-2 RdRp only the potential inhibitor of drugs or compounds in clinical trials with anti-HCV action. Among drugs tested, the potential of ribavirin is highlighted, which had also demonstrated potential antiviral action in the work of [88], but acting against the PLpro target (Figure 7b).

**Figure 8:**
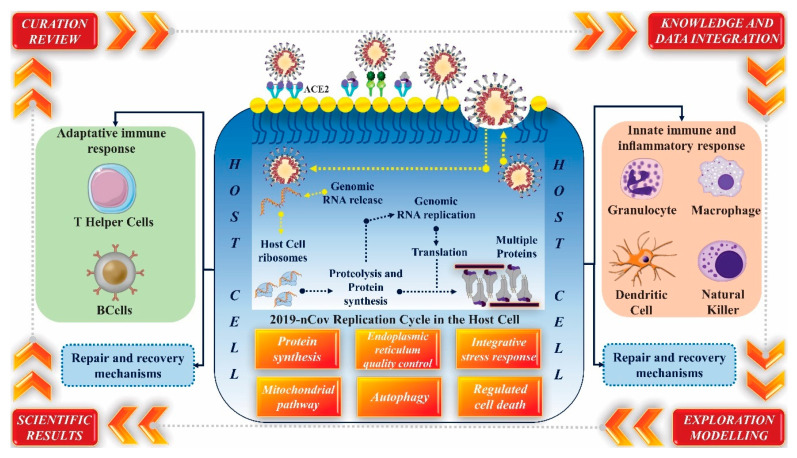
Overview of the functioning of the COVID-19 Disease Map: The map was created with a focus on the SARS-CoV-2 replication process, its interactions with host cells, immune system reactions, and repair mechanisms. According to the creators of the technology, the content inserted in the COVID-19 Disease Map is selected and reviewed from databases and knowledge continuously. This update is carried out according to the materials available in databases on the subject to support visual and computational exploration, as well as efforts to model diseases [137]. Note: This image was developed using the CorelDraw software (2017 Corel Corporation ID 410003).

Currently, a comprehensive and standardized repository of knowledge of the interaction mechanisms between SARS-CoV-2 and the host has been created. The repository is called COVID-19 Disease Map and was built with the contribution of experts using the results of published research, including bioinformatics data. This technology is a platform that contributes to the computational analysis of molecular processes involved in the 2019-nCoC input and replication interactions in the host. Besides, information about the immune system’s performance, recovery of host cells, and repair mechanisms can be obtained through the COVID-19 Disease Map [137]. Figure 8 demonstrates the objective and the initial layout of the map and its operating cycle.

A recent study involving the phylogenetic analysis of 160 complete genomes of COVID-19, researchers revealed three central variants distinguished by changes in amino acids, which were called variants A, B, and C, with A being the ancestral type according to the bat outgroup coronavirus. Viral strains A and C have been identified in many countries outside East Asia, that is, in Europe and the United States of America. In contrast, variant B is the most common type in East Asia, and there is no evidence that its ancestral genome spread outside East Asia without first undergoing mutations [138].

A phylogenetic analysis carried out in Turkey, with the first 30 genomes isolated from SARS-CoV-2 in this region, showed that the introduction of the virus in the country occurred before the first reported case of infection. The study demonstrated that the virus circulated in Turkey from several independent international introductions and revealed a hub for inland transmission [139].

Another phylogenetic analysis carried out recently sequenced nine viral genomes of patients with COVID-19 previously reported in Connecticut, United States. The research placed most of these genomes with viruses sequenced from Washington State. Interestingly, when the researchers combined the genomic data obtained in the study with national and international travel patterns, they found that domestic introductions probably drove the initial transmission of SARS-CoV-2 in Connecticut. This study provides evidence for the sustained and widespread transmission of SARS-CoV-2 in the USA and highlights the critical need for local surveillance [140].

Important research on the evolutionary and epidemiological dynamics of the current outbreak of COVID-19 analyzed 12 genomes of SARS-CoV-2 strains collected from China and 12 other countries with sampling dates between 24 December 2019 and 9 February 2020. The phylogenetic study results showed that the time to the most recent common ancestor was 2 November 2019, and the evolutionary rate of SARS-CoV-2 was 9.90 × 10^−4^ substitutions per site per year. These results highlight that the use of phylodynamic analyzes is crucial to provide insights into the possibilities of interventions to limit the spread of SARS-CoV-2 worldwide [141].

From studies like these, it is possible to suggest that phylogenetic networks developed with bioinformatics aid can also be used successfully to trace undocumented sources of COVID-19 infection. After that, cases can be reported and quarantined to prevent the disease’s recurrent spread worldwide [138].

## 6. Future Perspectives on Bioinformatics and Molecular Modeling as Prospecting Tools Against SARS-CoV-2

The contributions of bioinformatics and molecular modeling in elucidating essential targets for the planning and development of new drugs, and the analysis of already known compounds, support the search for safer and more effective treatments against SARS-CoV-2 infection. Identifying targets, planning interactions, understanding the structure-activity relationship, coupling, and molecular dynamics allow a projection of the mechanism of antiviral action in silico. An example of this is the prospect of using molecules of natural origin tested in vitro or in vivo and which showed significant antiviral activity. Therefore, the use of software to identify viral targets and the prediction of their interaction with the molecule under analysis is necessary so that that research can proceed safely and with a higher probability of success. The verification of the performance of these compounds through computational analysis can predict physical-chemical properties, which are related to pharmacodynamic and pharmacokinetic parameters, whose knowledge is crucial when it comes to the planning of new drugs [142]. Table 1 presents studies where the researchers tested natural compounds from plants and fungi against SARS-CoV-2. The table shows the results of these analyzes on viral replication. It provides an essential overview of the antiviral action of phytochemicals that can be studied in bioinformatics and molecular modeling for the design of new anti-SARS-CoV-2 therapies. In an attempt to mitigate the symptoms of COVID-19, accelerate recovery, and reduce the mortality rate, several known drugs are being tested against SARS-CoV-2, aiming at an antiviral action, the development of effective treatments, and prevention of infections by opportunistic microorganisms. The interaction of these drugs with proteins and viral receptors can be clarified through bioinformatics, which can identify the target and, with the support of molecular modeling, test the structural overlap, chemical interactions, and molecular coupling. Studies like these are fundamental to elucidate the antiviral action mechanism of the compounds, favoring the projection of molecular modifications that modulate the affinity and specificity of the drug and, consequently, its aspects of pharmacological potency and safety. Proposals for the repurposing of drugs known are a more reliable option for the development of an efficient and developed therapy with the urgency that the moment requires, to reduce the number of deaths [143]. Table 2 presents studies on the effects of using drugs already known, and therapies with combinations of drugs, and the results of these research on the clinical condition of that infected and viral replication. These studies serve as a basis for more accurate analyzes of the drug’s mechanism of action and the test outcome.

In the context of the COVID-19 pandemic, some studies and clinical reports describe the clinical worsening of some patients. The condition became known as cytokine storm or hypercytokinemia and is associated with increased mortality in infected patients. Among the proposed pharmacological strategies, is the use of anti-cytokine molecules, such as anti-interleukin drugs and inhibitors of IFN-γ and TNF-α (see Figure 4), capable of reducing the inflammatory process. The elucidation of the mechanisms associated with the action of immunomodulatory drugs on hypercytokinaemia is essential for the knowledge of safety regarding the use of these drugs, the best pharmacotherapeutic management, and the effectiveness to reduce the risk of death. Bioinformatics, when applied to the analysis of inflammatory mediators, is a tool capable of evaluating the virus’s performance and its ability to trigger an intense inflammatory response and how anti-cytokine molecules can normalize this condition. In this sense, bioinformatics aims to plan therapies that prevent the disease from worsening without causing immunodeficiency [144,145]. Table 3 shows the research results that used anti-cytokine compounds on respiratory function and inflammatory indexes in patients with COVID-19. The table reveals the clinical outcomes of the use of these substances, which can be investigated with the aid of bioinformatics and molecular modeling as a potential therapy against hypercytokinaemia resulting from SARS-CoV-2 infection, significantly reducing patient mortality.

It is crucial to mention that several of the drugs mentioned in this review have already received temporary regulatory approval. For example, remdesivir, hydroxychloroquine, chloroquine, and azithromycin are being used with consent in treatment protocols, depending on the country. The combination of these substances has also been used. Despite this, studies on its effectiveness in the different stages of SARS-CoV-2 infection are controversial, and additional research is needed to elucidate the efficacy of these substances in infected patients.

## 7. Conclusions

The use of molecular modeling at the early stages of searching for new bioactive compounds contributes to the faster development of new drugs at significantly reduced costs. Given the low number of compounds with known anti-SARS-CoV-2 activity and considering the availability of structural models of potential molecular targets of the virus, the strategy based on the structure of the molecular target has been further explored, in which molecular docking techniques play a crucial role in this planning method. The compound PubChem CID 444,745 showed the highest potential to inhibit SARS-CoV-2 proteases during studies in search of possible molecular targets against the virus.

About phytochemicals, the virtual libraries that store information about these compounds have already been extensively explored. Among the selected compounds, PubChem CID 11,610,052 and quercetin showed a strong interaction with 3CLpro from SARS-CoV-2. Also, Dihydrotansinone I interact strongly with spike glycoprotein and Cryptotanshinone, with proteins PLpro and 3CLpro. Marine compounds were also evaluated in silico against the main SARS-CoV-2 protease. The results identified different compounds as potential inhibitors of this protease, highlighting heptafuhalol A as the most promising.

Another virtual screening strategy that stands out among the research is the one that uses groups of drugs with antiviral action already approved for use in humans, characterizing a drug repositioning strategy. It was shown that digitoxin and zorubicin have a high potential for interaction with the SARS-Cov-2 spike glycoprotein. Another highlight was the potential for ribavirin to interact with the PLpro, lymecycline against 3CLpro, and valganciclovir against RNA-dependent RNA polymerase, essential proteins for the survival of SARS-CoV-2. Besides, the present review showed that the pemirolast is promising as an inhibitor of the action of spikes glycoproteins.

In addition to issues related to bioinformatics and molecular modeling, the details provided in the present review envision future points of consideration in the field of virology and medical sciences that will contribute to understanding the molecular mechanisms responsible for the pathogenesis and virulence of SARS-CoV-2 well as the development of the human acute severe respiratory syndrome. It is well established that the pathophysiology and lung injury caused by SARS-CoV-2 is related to innate immunity and factors such as the release of pro-inflammatory cytokines and the complement system action. Besides, we strongly emphasize that: (1) the complement system is an essential element of protective immunity against pathogens, but its excessive or unregulated activation can result in critical tissue damage and; (2) the viral S2 subunit can be an important target for future antiviral compounds. Thus, anti-S2 antiviral compounds may be potential treatments for SARS-CoV-2 infection. During this unprecedented period, we encourage scientists to actively contribute to understanding the role of the complement system in the development of COVID-19.

## Figures and Tables

**Table 1:** Compounds of natural origin with anti-SARS-CoV-2 activity.

Origin	Species	Compounds	Molecular Structures	Model Used	Doses and Route of Administration	Duration	Results	Ref.
Plant	*Nerium oleander*	Oleandrin	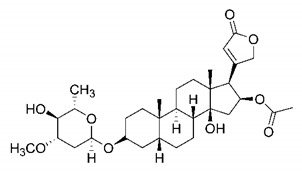	Vero cells	0.05 μg/mL	48 h	Viral replication reduction up to 78 times	[146]
					0.1 μg/mL		Viral replication reduction up to 100 times	
Plant	*Lonicera japonica*	Chlorogenic acid	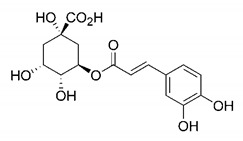	VeroE6 cells	76.4 µM	48 h	100% inhibition of SARS-CoV-2 3CLpro	[147]
					39.48 µM		IC_50_ for 3CLpro of SARS-CoV-2	
					20.2 µM		10% inhibition of SARS-CoV-2 3CLpro	
		Neochlorogenic acid	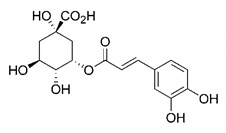	VeroE6 cells	49.2 µM	48 h	100% inhibition of SARS-CoV-2 3CLpro	
					10.4 µM		10% inhibition of SARS-CoV-2 3CLpro	
		Isochlorogenic acid A	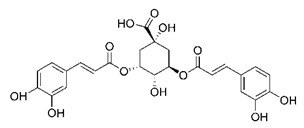	VeroE6 cells	77.0 µM	48 h	100% inhibition of SARS-CoV-2 3CLpro	
					18.9 µM		10% inhibition of SARS-CoV-2 3CLpro	
		Isochlorogenic acid B	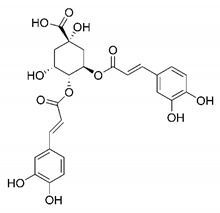	VeroE6 cells	52.4 µM	48 h	100% inhibition of SARS-CoV-2 3CLpro	
					26.3 µM		10% inhibition of SARS-CoV-2 3CLpro	
		Isochlorogenic acid C	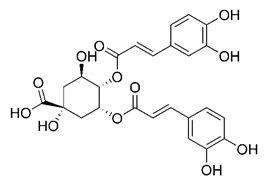	VeroE6 cells	78.2 µM	48 h	100% inhibition of SARS-CoV-2 3CLpro	
					18.4 µM		10% inhibition of SARS-CoV-2 3CLpro	
		1,3-Dicaffeoylquinic acid	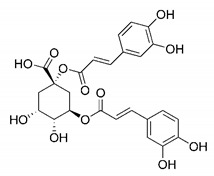	VeroE6 cells	87.3 µM	48 h	100% inhibition of SARS-CoV-2 3CLpro	
					27.8 µM		10% inhibition of SARS-CoV-2 3CLpro	
		Luteoloside	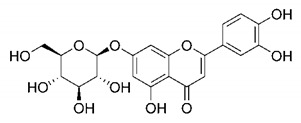	VeroE6 cells	65.4 µM	48 h	100% inhibition of SARS-CoV-2 3CLpro	
					14.8 µM		10% inhibition of SARS-CoV-2 3CLpro	
Plant	*Scutellaria baicalensis*	Baicalin	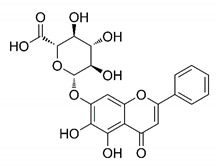	VeroE6 cells	97.6 µM	48 h	100% inhibition of SARS-CoV-2 3CLpro	[147]
					6.41 µM		IC_50_ for 3CLpro of SARS-CoV-2	
					68.9 µM		10% inhibition of SARS-CoV-2 3CLpro	
		Baicalein	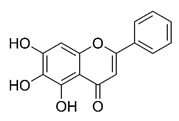	VeroE6 cells	99.4 µM	48 h	100% inhibition of SARS-CoV-2 3CLpro	
					0.94 µM		IC_50_ for 3CLpro of SARS-CoV-2	
					87.0 µM		10% inhibition of SARS-CoV-2 3CLpro	
		Scutellarein	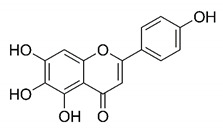	VeroE6 cells	101.6 µM	48 h	100% inhibition of SARS-CoV-2 3CLpro	
					3.02 µM		IC_50_ for 3CLpro of SARS-CoV-2	
					90.7 µM		10% inhibition of SARS-CoV-2 3CLpro	
		Scutellarin	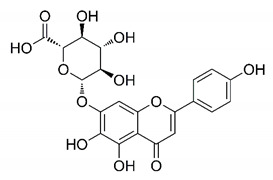	VeroE6 cells	76.8 µM	48 h	100% inhibition of SARS-CoV-2 3CLpro	
					18.9 µM		10% inhibition of SARS-CoV-2 3CLpro	
		Chrysin-7-O-β-D-glucuronide	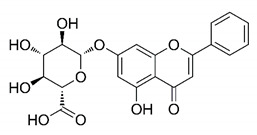	VeroE6 cells	50.6 µM	48 h	100% inhibition of SARS-CoV-2 3CLpro	
					24.2 µM		10% inhibition of SARS-CoV-2 3CLpro	
Plant	*Forsythia suspensa*	Forsythoside A	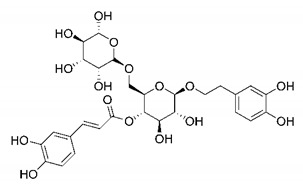	VeroE6 cells	95.3 µM	48 h	100% inhibition of SARS-CoV-2 3CLpro	[147]
					3.18 µM		IC_50_ for 3CLpro of SARS-CoV-2	
					70.5 µM		10% inhibition of SARS-CoV-2 3CLpro	
		Forsythoside B	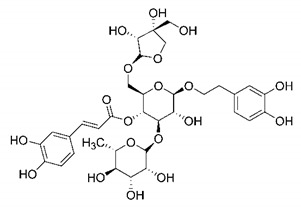	VeroE6 cells	101.4 µM	48 h	100% inhibition of SARS-CoV-2 3CLpro	
					2.88 µM		IC_50_ for 3CLpro of SARS-CoV-2	
					80.9 µM		10% inhibition of SARS-CoV-2 3CLpro	
		Forsythoside E	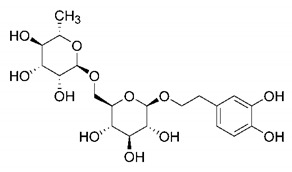	VeroE6 cells	96.6 µM	48 h	100% inhibition of SARS-CoV-2 3CLpro	
					6.68 µM		IC_50_ for 3CLpro of SARS-CoV-2	
					41.9 µM		10% inhibition of SARS-CoV-2 3CLpro	
		Forsythoside H	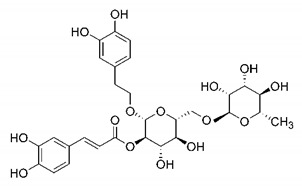	VeroE6 cells	99.3 µM	48 h	100% inhibition of SARS-CoV-2 3CLpro	
					10.17 µM		IC_50_ for 3CLpro of SARS-CoV-2	
					61.7 µM		10% inhibition of SARS-CoV-2 3CLpro	
		Forsythoside I	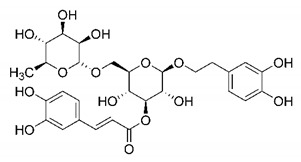	VeroE6 cells	95.9 µM	48 h	100% inhibition of SARS-CoV-2 3CLpro	
					5.47 µM		IC_50_ for 3CLpro of SARS-CoV-2	
					46.3 µM		10% inhibition of SARS-CoV-2 3CLpro	
		Isoforsythiaside	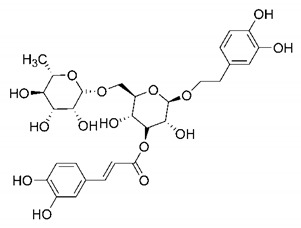	VeroE6 cells	94.4 µM	48 h	100% inhibition of SARS-CoV-2 3CLpro	
					5.85 µM		IC_50_ for 3CLpro of SARS-CoV-2	
					46.8 µM		10% inhibition of SARS-CoV-2 3CLpro	
		Acteoside	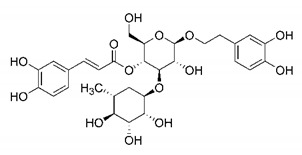	VeroE6 cells	97.0 µM	48 h	100% inhibition of SARS-CoV-2 3CLpro	
					34.6 µM		10% inhibition of SARS-CoV-2 3CLpro	
Fungus	*Colletotrichum gloeosporioides*	Phillyrin	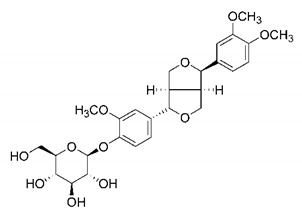	VeroE6 cells	10 µM	48 h	7,8% inhibition of SARS-CoV-2 3CLpro	

**Table 2:** Compounds of synthetic origin with anti-SARS-CoV-2 activity.

Compounds	Molecular Structures	Model Used	Doses and Route of Administration	Duration	Results	References
Colchicine	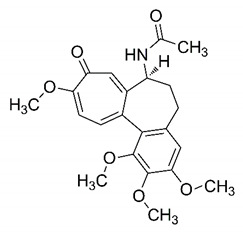	Humans	1.5 mg followed by 0.5 mg after 60 min and maintenance doses of 0.5 mg twice daily	3 weeks	It had significantly improved in the time of clinical deterioration	[148]
			1 mg/day	21 days	Best survival rate	[149]
Remdesivir	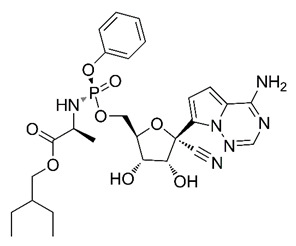	Vero E6 cells	1.65 μM	48 h	Reduced inhibitory activity on viral replication	[150]
		Calu3 2B4 human lung cells	0.28 μM	72 h	Strong inhibition of SARS-CoV-2 replication	
		Human airway epithelial cells	0.01 μM	48 h	Strong inhibition of SARS-CoV-2 replication	
		Mice	25 mg/kg	5 days	Drastic reduction of viral load in the lung	
		Humans	200 mg on day 1 followed by 100 mg on days 2 to 10, intravenously	10 days	No statistically significant clinical benefits were observed	[151]
		Humans			Clinical improvement and fewer adverse effects	[152]
Lopinavir + Ritonavir *	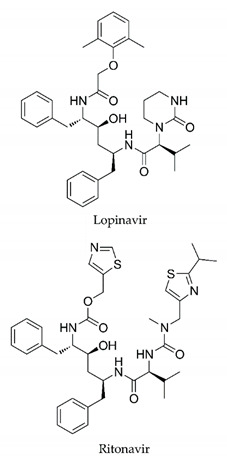	Humans	400 mg + 100 mg	14 days	No benefit was seen	[153]
		Humans	800 mg per day	6 days	Absence of viral clearance	[154]
IFN-β-1b + Lopinavir + Ritonavir + Ribavirin *	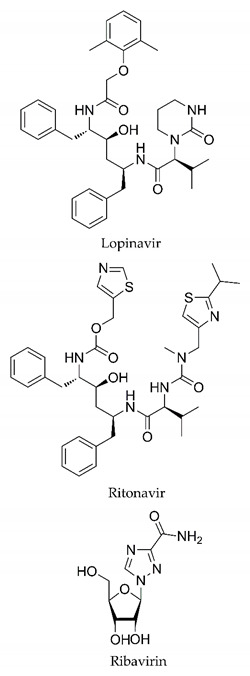	Humans	8 million international units + 400 mg + 100 mg + 400 mg	14 days	Relief of symptoms and reduction of hospital stay	[155]
IFN-β-1a	*	Humans	44 µg subcutaneously every two days	10 days	Improved viral clearance and faster recovery speed	[156]
Hydroxychloroquine	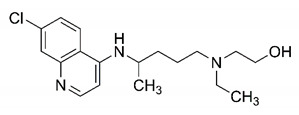	Humans	1200 mg daily for three days and a maintenance dose of 800 mg daily	Two or three weeks depending on the severity of the condition	No virus elimination benefits were observed	[157]
		Humans	400 mg per day	5 days	Improved patient prognosis	[158]
		Humans	600 mg per day	6 days	Reduction/disappearance of viral load	[159]
		Humans	400 mg HCQ twice daily on day 1, followed by 200 mg twice daily on days 2 to 5	5 days	Significant reduction in mortality	[160]
		Vero cells	0.72 μM	48 h	Significant antiviral activity	[161]
			6.14 μM	24 h	Insignificant antiviral activity	
		VeroE6 cells	4.06 μM	48 h	Significant antiviral activity	[162]
Azithromycin	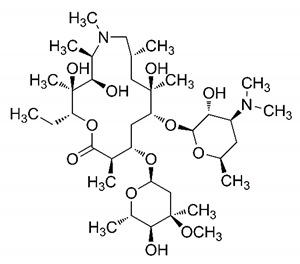	Humans	500 mg of AZTM once daily on day 1, followed by 250 mg once daily for the next 4 days	5 days	No benefit was seen	[160]
Hydroxychloroquine + Azithromycin *	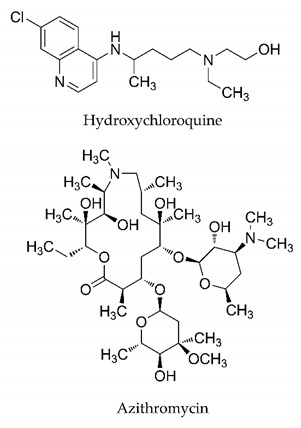	Vero E6	1 μM of HCQ + 5 μM of AZTM or + 10 μM of AZTM	60h	No significant results were observed	[163]
			2 μM of HCQ + 5 μM of AZTM or + 10 μM of AZTM			
			5 μM of HCQ + 5 μM of AZTM		Relative viral inhibition of 97.5% and 99.1%	
			5 μM of HCQ + 10 μM of AZTM			
		Humans	400 mg HCQ × 2 on day 1, followed by 200 mg × 2 on days 2 to 5 + 500 mg of AZTM on day 1, followed by 250 mg the next 4 days	5 days	Significant reduction in mortality	[160]
		Humans	200 mg HCQ × 3/day + 500 mg of AZTM on day 1 followed by 250 mg/day the next four days	HCQ for ten days + AZTM for 5 days	Administration prior to the occurrence of complications was associated with a reduction in the mortality rate	[164]
		Humans	600 mg HCQ + 500 mg AZTM, followed by 250 mg AZTM on the following days	6 days	Absence of viral clearance	[154]
		Humans	400 mg HCQ, twice on day 1, followed by 200 mg twice for the next 4 days + 500 mg of AZTM for 3 days	HCQ for 5 days + AZTM for 3 days	No security problems were found using the combination	[165]
		Humans	200 mg HCQ × 3/day + 500 mg AZTM on day 1, followed by 250 mg for the next 4 days orally	HCQ for 10 days + AZTM for 5 days	Improvement in clinical results and faster reduction of viral load	[166]
Chloroquine Diphosphate	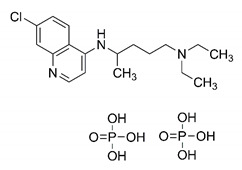	Humans	600 mg twice daily	10 days	Higher lethality rate	[167]
			450 mg twice daily	4 days	No benefit was seen	
		Vero cells	5.47 μM	48 h	Insignificant antiviral activity	[161]
			23.90 μM	24 h		
		VeroE6 cells	2.71 μM	48 h	Insignificant antiviral activity	[162]
Ivermectin	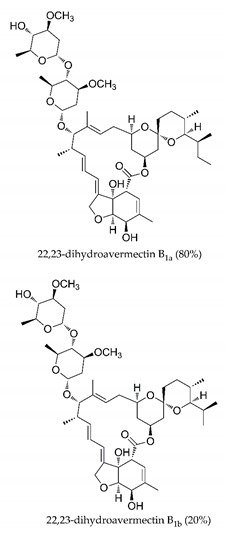	Vero-hSLAM cells	5 μM	48 h	Reduction of viral replication approximately 5000 times	[168]
Nafamostat mesylate	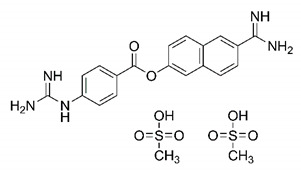	Calu-3 cells	10 nM	5 days	Potent inhibition of SARS-CoV-2 fusion mediated by protein S and consequent inhibition of infection	[169]
		VeroE6/TMPRSS2 cells	30 μM	3 days		
Cenicriviroc	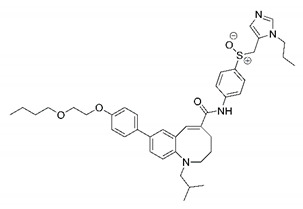	VeroE6/TMPRSS2 cells	40 μM	3 days	Inhibition of viral replication and control of excessive immune response	[170]
Baricitinib	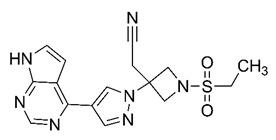	Humans	4 mg per day	2 weeks	Reduction of inflammatory indices and clinical improvement	[171]

AZTM: Azithromycin; HCQ: Hydroxychloroquine; * Associated therapy.

**Table 3:** Anti-cytokine compounds with anti-SARS-CoV-2 activity.

Compounds	Model Used	Doses and Route of Administration	Duration	Results	References
Tocilizumab	Humans	80 to 600 mg, depending on the severity of the condition	38 days	Reduces risk of cytokine storms in patients with severe COVID-19	[172]
		400 mg intravenously	3 days	Cytokine release syndrome even after treatment	[173]
		560 mg for 2 days, followed by 700 mg the next day intravenously			
		400 mg	_	Clinical improvement and lower mortality	[174]
		400 mg to 800 mg according to the severity of the condition, intravenously	Single dose, 1 h infusion	Immediate improvement of symptoms and normalization of inflammatory indices after 5 days	[175]
		8 mg/kg intravenously every 12 h and a third infusion 24 h later	2 days	Improvement of clinical and respiratory condition	[176]
		8 mg/kg intravenously	6 days	Improvement in respiratory and laboratory parameters	[177]
		8 mg/kg once daily	2 days	The treatment associated with hemoadsorption, improved gas exchange and reduced levels of inflammatory mediators	[178]
Sarilumab	Humans	400 mg intravenously	10 days	Treatment was associated with faster recovery	[179]
			5 days	Reduction of inflammation and rapid recovery	[180]
		200 mg	_	Clinical improvement and lower mortality	[174]
Anakinra	Humans	200 mg intravenously, followed by 100 mg every 6 h subcutaneously	_	Progressive improvement in respiratory function and Marked reduction in inflammatory markers	[181]
		200 mg every 8 h intravenously	7 days	Improved respiratory function	[182]
		300 mg once daily intravenously, followed by 100 mg once daily subcutaneously	300 mg for 4 days + 100 mg until hospital discharge		
		5 mg/kg twice a day intravenously	10 days	Reduction of systemic inflammation and progressive improvement in respiratory function	[183]
		100 mg twice daily subcutaneously	7 days	Absence of significant clinical or anti-inflammatory effects	
Infliximab	Humans	10 mg/kg	2 days	Interruption of the systemic inflammatory response in a patient with Crohn’s disease and COVID-19	[184]
Eculizumab	Humans	4 weekly infusions of 900 mg	4 weeks	Clinical improvement in the first 48 h after the first administration	[185]
